# Multiorgan Dysfunction With Severe Cardiac Injury Secondary to Septic Cellulitis Due to Vibrio parahaemolyticus

**DOI:** 10.7759/cureus.31673

**Published:** 2022-11-19

**Authors:** Anh Tuan Mai, Duy Chung, Luan Ngo, Kim Huyen Huynh, Ly T Dinh

**Affiliations:** 1 Critical Care Medicine, Emergency Medicine and Clinical Toxicology, University of Medicine and Pharmacy at Ho Chi Minh City, Ho Chi Minh City, VNM; 2 Internal Medicine, Pham Ngoc Thach University of Medicine, Ho Chi Minh City, VNM; 3 Intensive Care, Cho Ray Hospital, Ho Chi Minh City, VNM

**Keywords:** septic cardiomyopathy, multi-organ dysfunction syndrome, vibrio parahaemolyticus, septic shock, cellulitis

## Abstract

*Vibrio parahaemolyticus* infection commonly manifests as gastroenteritis, including diarrhea, abdominal pain, nausea, vomiting, and fever. Although uncommon, *V. parahaemolyticus* has also been associated with wound infection and septic shock. These two manifestations have not been well-reported in medical literature, yet may yield a high risk of death, thus requiring emergent interventions. We present a case of a 42-year-old patient who developed septic shock secondary to a bullous necrotic wound and diarrhea due to *V. parahaemolyticus*. Multi-organ dysfunction syndrome with extreme cardiac injury developed very early in the course of the disease, prompting ICU admission and management with antibiotics, fluid resuscitation, vasopressors, blood purification, and surgical debridement. The treatment achieved a good clinical outcome. To the best of our knowledge, this is the first case report of *V. parahaemolyticus*-induced cardiomyopathy. *V. parahaemolyticus* should be considered as one of the causative agents in patients with sepsis due to cellulitis, particularly in patients with a suggestive history, such as exposure to seawater or eating seafood.

## Introduction

*Vibrio parahaemolyticus* is a gram-negative, facultative halophile, and non-spore-forming bacterium. Similar to other Vibrio species, *V. parahaemolyticus* exists as a free-living bacterium in estuarine or marine environments and is a common seafood contaminant. Since the mid-1990s, there has been a global increase in the incidence of *V. parahaemolyticus* infections that are associated with the appearance of a new pandemic variant, *V. parahaemolyticus* O3:K6 [1]. This pathogen continues to be found, discussed, and reported with increased incidence. Typically, it is associated with gastroenteritis, which clinically manifests as diarrhea, abdominal cramps, nausea, vomiting, and fever [2]. In addition, wound infections due to *V. parahaemolyticus* occasionally have been reported and associated with exposure to estuarine waters [3]. The infection can be extremely severe in the setting of liver disease, alcoholism, or diabetes. *V. parahaemolyticus* can also cause septicemia, particularly in individuals with underlying liver disease. These two manifestations are rare and have not been well-reported so far [4-5]. We report the first case of *V. parahaemolyticus*-induced cardiomyopathy and multiple organ dysfunction.

## Case presentation

A 42-year-old male patient who is a sea fisherman, with no significant past medical history except for chronic alcohol abuse, was brought to a local hospital’s emergency room presenting with vomiting and diarrhea. He had not seen a physician in 10 years. Three hours prior to admission, he had a party at the beach, where he consumed several alcoholic drinks and squid salad. Upon returning home, he discovered a scratch on his right calf, which developed into dark blisters over the next four hours. He developed nausea, non-bloody vomiting, several episodes of watery diarrhea, and shortness of breath without hematochezia or melena.

**Figure 1:**
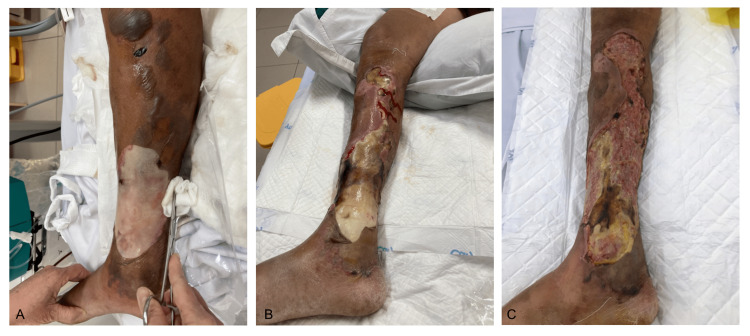
The progression of initial skin lesions (A) on admission at intensive care unit, (B) a week after admission, (C) 10 days after admission

Upon admission, the skin lesion spread to the surrounding areas forming enlarging blisters, and some of them ruptured (Figure 1). The patient’s hemodynamic status rapidly deteriorated, and fluid resuscitation, vasopressors, and broad-spectrum antibiotics, including ciprofloxacin and ceftazidime, were administered. Serum laboratory results were as follows: leukocyte of 27.09 G/L with 93.8% of neutrophil, serum creatinine of 4.4 mg/dl, and troponin I of 1.42 ng/ml. Despite fluid resuscitation and vasopressor administration, heart rate and mean arterial pressure (MAP) were 130-140 bpm and 60-65 mmHg, respectively. The patient was then transferred to our institute due to hemodynamic instability.

While in the emergency department, the patient was in septic shock with severe leukocytosis, metabolic acidosis, hyperlactatemia, oliguria, and respiratory distress warranting supplemental oxygen via rebreather face mask to maintain pulse oxygen saturation of 90%-94% (Table 1). A MAP greater than 65 mmHg was maintained with fluid and norepinephrine of 8-40 mcg/min. An extending ulcer (15 cm x 10 cm) with several darkened bullae, fibrinous and malodorous coating, without bone or joint involvement, was observed at the lower one-third of the medial right leg just above the medial malleolus (Figure 1). An orthopedic surgeon was consulted, and an anterior-posterior X-ray of the tibia and fibula was obtained to rule out osteomyelitis. Broad-spectrum antibiotics and supplemental oxygen were administered. Sequential Organ Failure Assessment score was used to assess severity. The score of 14 indicated a high mortality rate, and consequently the patient was sent to the ICU.

**Table 1:** Laboratory results on admission at our hospital RBC = red blood cell; MCV = mean corpuscular volume; WBC = white blood cell; PLT = platelet; PCT = procalcitonin; ALT = alanine transaminase; AST = aspartate transaminase; BUN = blood urea nitrogen; CK-MB = creatine kinase-MB; FiO2 = fraction of inspired oxygen; pCO2 = partial pressure of carbon dioxide; pO2 = partial pressure of oxygen; HCO3 = bicarbonate

Parameter	Value	Units	Reference values
RBC	3.55	M/L	3.8-5.5
Hemoglobin	11.9	g/dL	12.0-17.0
MCV	103.3	fL	78-100
WBC	23.5	G/L	4-11
Neutrophil	21.3	G/L	1.8-8.25
PLT	95	G/L	200-400
PCT	290	ng/mL	<0.5
Venous Lactate	4.39	mmol/L	0.5-2.2
Bilirubin			
Total	2.19	mg/dL	0.2-1.0
Indirect	1.75	mg/dL	0.2-0.8
Liver enzymes			
AST	3971.4	U/L	9-48
ALT	778.8	U/L	5-49
BUN	45.5	mg/dL	7-20
Serum creatinine	3.43	mg/dL	0.7-1.5
Serum sodium	137	mmol/L	135-150
Serum potassium	3.6	mmol/L	3.5-5.5
Serum chloride	106	mmol/L	98-106
CK-MB	144	ng/mL	<5.2
Troponin I	>50	ng/mL	<0.2
Serum IgG	641	mg/dL	700-1600
Arterial Blood Gas			
pH	7.17		
pO_2_	93	mmHg	
pCO_2_	23.5	mmHg	
FiO_2_	80%		
HCO_3_	8.7	mmol/L	

**Figure 2:**
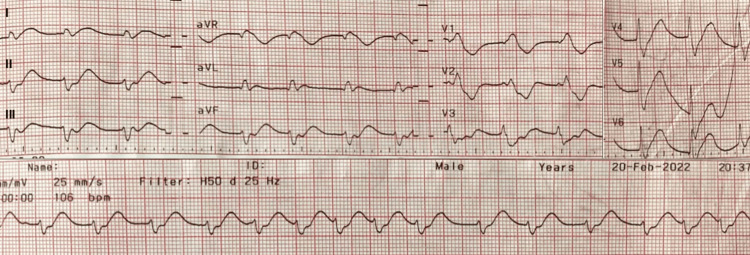
ECG on intensive care unit admission showed atrial fibrillation and QRS alterations

In the ICU, a diagnosis of septic shock secondary to cellulitis complicated by multiple organ dysfunction was established according to the Third International Consensus Definitions for Sepsis and Septic Shock [6]. The initial skin lesion expanded in size and depth, increasing from 15% to 30% of the right calf. All blisters burst and exposed necrotic tissue. Antibiotics were transitioned into intravenous meropenem, levofloxacin, and vancomycin, and the patient was intubated due to altered mental status and respiratory distress. At that time, we noted severe cardiac injury with highly elevated creatine kinase (CK)-MB and troponin I (Table 1). Electrocardiography revealed atrial fibrillation (AF), prolonged QRS duration (120 milliseconds), attenuated QRS amplitude, and right bundle branch block (Figure 2). Echocardiography showed global left ventricular hypokinesia with an ejection fraction (EF) of 29% by the Simpson method and mild dilated right ventricle with tricuspid annular plane systolic excursion of 14 mm. The Pulse Index Contour Continuous Cardiac Output system was implemented for measuring continuous cardiac output (CO). The patient’s hemodynamic profile during the ICU admission showed a heart rate of 113 bpm, arterial pressure of 139/60 mmHg, CO of 8.64 liters/minute, systemic vascular resistance (SVR) of 580 dyn.sec.cm-5.m2, and extra vascular lung water index of 13.2 ml/kg. A diagnosis of sepsis-induced cardiomyopathy (SCM) over cardiac ischemia was established. Terlipressin was added as the second vasopressor due to inadequate MAP and severe vasodilation. In addition to these medications, continuous venovenous hemodiafiltration using oXiris membrane (Baxter, Deerfield, IL, USA) was indicated due to multiple organ dysfunction, including oliguric acute renal failure (ARF) and severe hypotension. Wound culture revealed *V. parahaemolyticus* sensitive to current antibiotics (Table 2).

**Table 2:** Culture and antibiogram

Culture Results	
Collection sample	Right leg wound discharge
Specimen	Swab
Isolated Organism	Vibrio parahaemolyticus
Antibiogram	
Antimicrobial agent	Interpretation
Ceftazidime	Sensitive
Cefotaxime	Sensitive
Cefepime	Sensitive
Meropenem	Sensitive
Ciprofloxacin	Sensitive
Levofloxacin	Sensitive
Trimethoprim/Sulfamethoxazole	Sensitive
Imipenem	Sensitive
Ampicillin	Resistant

**Figure 3:**
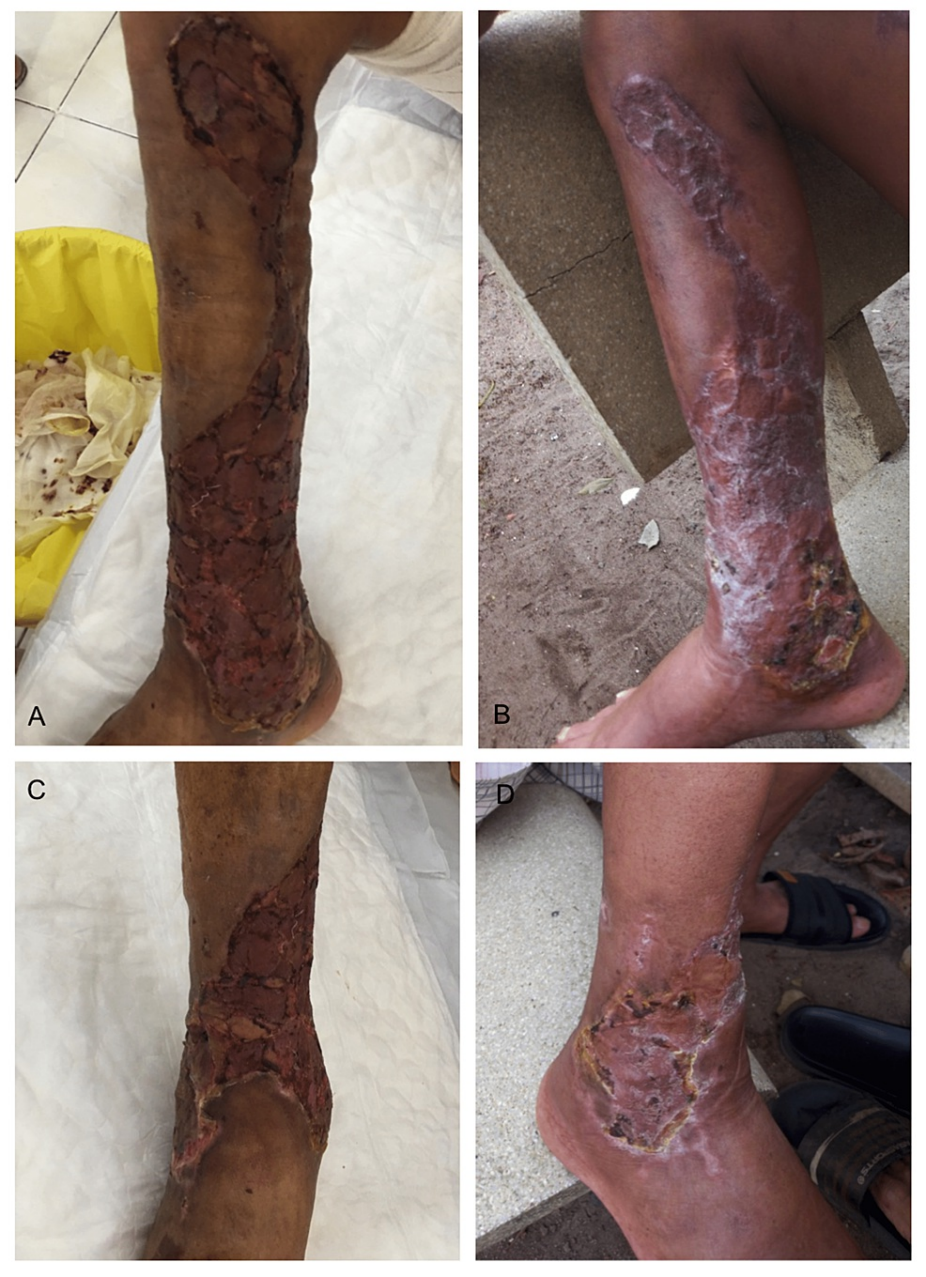
Appearance of the healed wound following skin graft (A,C) before hospital discharge, (B,D) three weeks after hospital discharge

Vasopressors were weaned off after three days. Blood purification was transitioned into intermittent hemodialysis (IHD) to address persistent oliguric ARF. The patient started urination after three episodes of IHD; however, the hospital course was complicated by an episode of ventilator-associated pneumonia (VAP) due to multidrug-resistant *Acinetobacter baumannii.* Colistin was initiated according to the antibiogram of the tracheal sputum culture. Procalcitonin levels trended down from 290 ng/ml (ICU day 1) to 5 ng/ml (ICU day 10) and 0.6 ng/ml (ICU day 18). Local wound care in the ICU consisted of daily cleaning and hydrophilic dressing. Surgical debridement of the necrotic wound was performed on ICU day 12 as soon as the patient’s cardiorespiratory status was adequately maintained with minimal vasopressors and ventilator support. Necrotic skin and subcutaneous fat tissue were removed, and the wound was thoroughly irrigated using normal saline. No necrotizing fasciitis, bone or tendon involvement was noted. The patient remained stable and was transferred to the burn unit on day 16. Skin graft surgery was performed twice on days 28 and 35. He was discharged after recovery from the operation. One month after discharge, the wound was completely healed with a satisfactory granulation of wound base (Figure 3).

## Discussion

*V. parahaemolyticus*, like other *Vibrio *spp., exists as a free-living bacterium in estuarine or marine environments, interacting with zoo- and phytoplankton or marine plants (algae, coral, and sponges) and animals (crustaceans, squid, and fish) [4]. It is a common seafood contaminant. The number of bacteria in the environment (and in oysters) tends to increase as water temperature rises, making this microorganism particularly susceptible to the effect of global warming [7]. *V. parahaemolyticus* survives at 5-45°C and achieves substantial growth when seawater temperatures are over 14-19°C, which explains why this pathogen is prevalent in summer and autumn [8]. The bacterial count may also be influenced by salinity, and higher counts have been observed in regions of lower salinity [9]. At present, a third of reported *Vibrio* infections are due to this pathogen [8].

*Vibrio* spp. are classified into two groups: cholera and non-cholera infections. While *V. cholerae* causes gastroenteritis almost exclusively, non-cholera *Vibrio *spp. can manifest in many clinical presentations, including gastroenteritis, skin infection, and sepsis [4,10-11]. *V. alginolyticus*, *V. damsela*, and *V. vulnificus* usually cause skin infections. Sepsis is more frequent among patients affected by non-O1 *V. cholerae* and *V. vulnificus*, while it is uncommon in patients infected by *V. alginolyticus*, *V. fluvialis*, *V. hollisae*, *V. mimicus*, and *V. parahaemolyticus* [4,10-11]. The pathogenicity of *V. parahaemolyticus* depends on the production of thermostable direct hemolysin (*V. parahaemolyticus*-TDH), which is responsible for β-hemolysis. Most strains of *V. parahaemolyticus* isolated from the environment or seafood, in contrast to clinical strains, do not produce TDH [12]. *V. parahaemolyticus*-TDH-related hemolysin is the second group of hemolysins that can be found in certain clinical isolates of *V. parahaemolyticus*. β-hemolytic strains are considered to be pathogens [13].

Since the first report of *V. parahaemolyticus* was published in 1950 [14], *V. parahaemolyticus* infections have normally manifested as gastroenteritis. In recent years, an increase in wound infections has been observed. Skin infections due to *V. parahaemolyticus*, predominantly cellulitis, occur through the exposure of acute or chronic wounds to saltwater. Initially, patients almost always report severe pain in the involved body part. Other skin manifestations are erythematous indurated plaques with hemorrhagic bullae, pustules, petechiae, necrosis, and ulcers [4-5]. Necrotizing fasciitis induced by *V. parahaemolyticus* has been rarely reported in the literature, compared to that induced by *V. vulnificus* [15]. Wound infections associated with exposure of wounds to estuarine waters are generally mild. However, for patients with underlying conditions, such as liver disease, alcoholism, or diabetes, cellulitis can be severe, with a reported case fatality rate of 3% [2,10]. Furthermore, the development of sepsis in patients with *V. parahaemolyticus* has become more common, especially in the presence of predisposing factors similar to those of cellulitis. These conditions strongly favor the development of *V. parahaemolyticus* septicemia in 5% of cases, which is much less frequent than sepsis caused by *V. vulnificus* (>30% of cases). Symptoms (high fever, chills, myalgia, and pain in the lower extremities) occur with an abrupt onset within 7-14 days after contact [4-5]. In a report of 17 patients with *V. parahaemolyticus* septicemia, the case fatality rate was 29% [2].

While gastroenteritis caused by *V. parahaemolyticus* tends to be mild and self-limited, wound infection due to this pathogen requires systemic antibiotic therapy in addition to usual local care, such as debridement and application of antiseptic products. Based on clinical trials in cholera and in vitro susceptibility data, doxycycline is a reasonable antibiotic choice; reasonable alternatives include fluoroquinolones and macrolides. Duration of therapy is dictated by clinical response; patients with mild wound infections who do not have any significant underlying diseases generally respond well to local care and oral antibiotics. Most patients respond to 5 to 7 days of antibiotics [4,5].

Our patient’s occupation suggests that his skin lesion would have been exposed to seawater and become contaminated. His past medical history of chronic alcohol abuse, together with macrocytic anemia identified on admission, suggests malnutrition status and previous liver injury, which may explain the mildly low serum immunoglobulin and severe progression of the disease [16].

**Figure 4:**
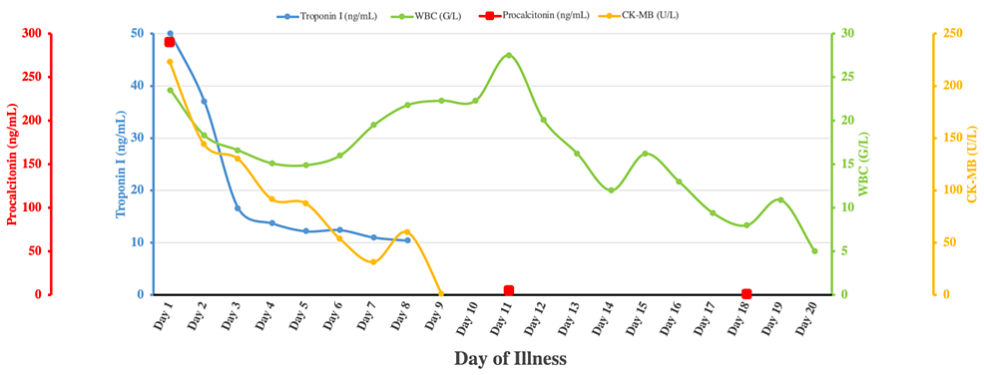
Progression of disease course WBC = white blood cell; CK-MB = creatine kinase-MB

Cardiac injury developed very rapidly and early in the course of the disease. The patient’s ECG changes, cardiac performance on echocardiography, and hemodynamic profile were consistent with SCM. The commonly reported alterations on ECG of septic shock and septic cardiomyopathy are new-onset AF, decreased QRS amplitude, increased QRS duration, and bundle branch block, which returns to baseline following recovery from septic shock [17]. This was seen in our patient. Echocardiography is considered the gold standard for the diagnosis of SCM, and the findings consist of left ventricular systolic and diastolic dysfunction and right ventricular dysfunction with or without reduced CO [18] were confirmed in our patient. Global longitudinal strain may be a more sensitive and specific parameter, and a value of less than 20% highly suggests SCM [18]. Unfortunately, we did not perform speckle tracking echography due to the unavailability of the dedicated device. The decreased EF and severely decreased SVR in our patient may indicate ventriculo-arterial coupling, a distinguishing feature in SCM. The significantly reduced SVR may falsely elevate the measured CO due to mathematical coupling, which can explain the impairment of intrinsic cardiomyocyte contractility despite the high measured CO in our patient [18]. The dramatic increase in serum troponin I in our patient is likely multifactorial and can be associated with myocardial membrane leakage and acute kidney injury. In addition, the trending down of leukocyte and procalcitonin, together with CK-MB and troponin I, and the disappearance of initial ECG alterations indicate the improvement of cardiac injury following the resolution of infection, which makes the diagnosis of septic cardiomyopathy very likely (Figure 4). To the best of our knowledge, this is the first case report that describes cardiomyopathy due to *V. parahaemolyticus* infection.

Nosocomial infection and the emergence of multidrug-resistant pathogens have been major issues in our institute for the past 10 years. This patient developed an episode of VAP due to multidrug-resistant *Acinetobacter baumannii* after partial improvement from the previous infection, which prolonged the ventilator-, ICU- and hospital length of stay and may affect the patient’s long-term prognosis.

## Conclusions

Even though the majority of interactions with *Vibrio *spp. are benign, pathogenic *Vibrio *spp. can cause severe infections in humans. Septic shock and cellulitis due to *V. parahaemolyticus *have become more frequent presentations and can be life-threatening, especially in patients with predisposing factors. The treatment is consistent with Surviving Sepsis Campaign guidelines including systemic antibiotic therapy, source control such as necrosis debridement, and supporting therapy. *V. parahaemolyticus* should be considered one of the causative agents in patients with sepsis due to cellulitis, particularly in patients with a suggestive history, such as exposure to seawater or eating seafood.
